# Interferometric image scanning microscopy enables label-free super-resolution imaging of live cells

**DOI:** 10.1038/s41377-026-02316-3

**Published:** 2026-05-21

**Authors:** Qianxi Liang, Wei Ren, Peng Xi

**Affiliations:** Department of Biomedical Engineering, National Biomedical Imaging Center, College of Future Technology, Peking University, 100871 Beijing, China

**Keywords:** Super-resolution microscopy, Phase-contrast microscopy

## Abstract

By implementing the super-resolution principles of image scanning microscopy in coherent imaging, Küppers et al. introduced interferometric image scanning microscopy, achieving 120 nm label-free lateral resolution with minimal phototoxicity and offering a robust tool for long-term observation of intracellular dynamics.

## Abstract (web summary)

Küppers et al. introduced interferometric image scanning microscopy (iISM), achieving 120 nm label-free super-resolution imaging for long-term live-cell observation with minimal phototoxicity.

## Graphical abstract

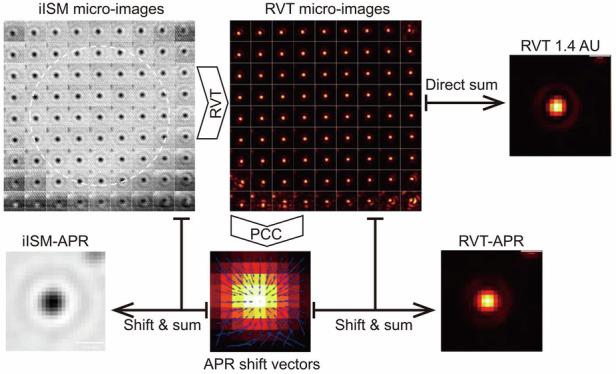

A fundamental challenge in microscopy remains the inherent trade-off between high spatiotemporal resolution, signal-to-noise ratio (SNR), and biocompatibility^1^. To address this, Küppers et al. recently reported interferometric image scanning microscopy (iISM) in *Light: Science & Applications*, which achieves label-free, super-resolution imaging of fine structures within live cells at an extremely low light dose (Fig. 1)^2^.

**Fig. 1:**
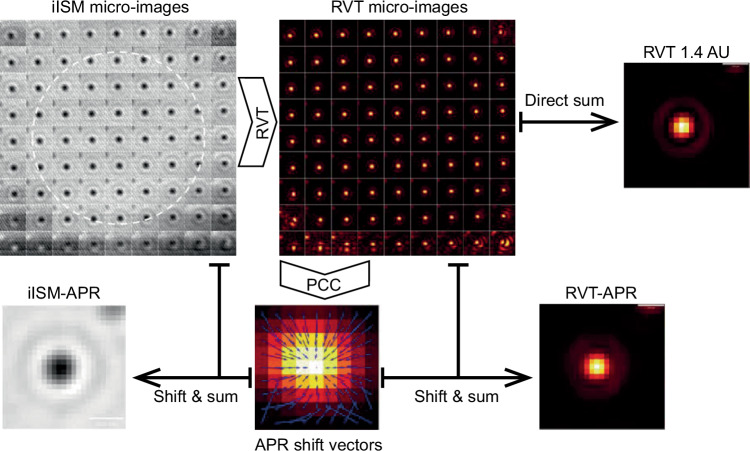
The workflow of iISM reconstruction and a comparison of the PSF before and after reconstruction

Image scanning microscopy (ISM) serves as a bridge connecting traditional confocal microscopy and super-resolution imaging. By utilizing an array detector to capture off-axis photons that would otherwise be blocked by the confocal pinhole, ISM applies pixel reassignment (PR) for spatial correction. Since this approach avoids the loss of photon flux, it pushes the resolution to the confocal limit while maintaining a high SNR^3,4^. However, traditional ISM frameworks, whether implemented through optical hardware or digital algorithms such as adaptive pixel reassignment (APR), are fundamentally predicated on an incoherent imaging model, where the final image is a linear superposition of intensities. This intensity-based paradigm faces significant challenges when integrated with coherent imaging techniques like interferometric scattering microscopy (iSCAT). In such coherent regimes, the system follows the principle of complex amplitude superposition, where phase information encoded within the intensity images leads to phase flips, rendering traditional ISM algorithms that rely on intensity correlations ineffective. Table 1 offers a comparative summary of various ISM reconstruction methods.

**Table 1:** Comparative summary of different ISM reconstruction methods

Method	Core mechanism	Limitation	References
Optical PR	Utilizing microlens arrays for beam shrinking or rescan optics to expand focal spot mapping.	High system complexity and implementation costs.	^5–8^
Conventional PR	Directly shifting pixel signals by a factor of 1/2 toward the optical axis.	Inability to correct system aberrations; ignores the effects of the Stokes shift.	^9,10^
APR	Utilizing cross-correlation to estimate displacement vectors directly from micro-images.	Dependent on the similarity of image intensities.	^11^
DPA-PR	Extending the APR to non-descanned or multifocal optical paths for spot movement.	Requires precise estimation of multifocal array parameters.	^12^
Inverse problem & deep learning	Based on optical field inversion or data-driven learning models.	Inverse methods are sensitive to physical model deviations; deep learning depends on high-quality ground truth for training.	^13–15^
RVT-APR (This work)	Utilizing RVT to transform interferometric images into intensity images that reflect centrosymmetry.		^2^

To overcome this challenge, the authors developed a novel iISM system that synergistically combines the advantages of iSCAT and ISM. The former enables high-sensitivity, label-free detection of nanostructures via the interference between scattered and reference light, while the latter enhances spatial resolution beyond the diffraction limit by finely sampling the focal spot with an array detector and applying reconstruction algorithms. For hardware implementation, the system adopts a confocal reflection geometry, utilizing an sCMOS camera to record complete micro-images. Addressing the issue where linearly polarized light often leads to an anisotropic point spread function (PSF) in interferometric imaging, the authors specifically introduced circularly polarized illumination. This ensures the rotational symmetry of the interferometric PSF and minimizes measurement artifacts, thereby establishing a solid physical foundation for subsequent high-precision image reconstruction.

Algorithmically, the authors proposed an enhanced APR workflow based on the radial variance transform (RVT). The process first employs RVT to convert interferometric micro-images containing phase information into intensity maps that reflect local symmetry, thereby simulating incoherent imaging characteristics. Building on this transformation, phase cross-correlation (PCC) is performed to extract precise displacement vectors, which are subsequently mapped back to the original interferometric data for sub-pixel signal reassignment. This integrated hardware–software strategy enables label-free, super-resolution imaging with a high SNR. Experimental data demonstrate that iISM achieves a lateral resolution of ~120 nm. Benefiting from the dual advantages of coherent imaging and ISM, iISM yields a contrast-to-noise ratio of up to 38 at the same photon flux. This performance represents an improvement of approximately 4-fold and 3-fold over traditional closed-pinhole (~10) and open-pinhole (~14) confocal modes, respectively, while significantly suppressing background noise.

In live-cell imaging, iISM demonstrates exceptional performance. The system requires an ultra-low incident power of only 0.5 µW, which is ~10 times lower than that of comparable confocal iSCAT techniques. This minimal dosage allows for the clear resolution of mitochondria, endoplasmic reticulum tubules, and actin filaments within COS-7 cells without inducing visible phototoxicity during long-term observation. Notably, the positive and negative interference contrasts in iISM images directly encode the nanometer-scale axial positions of organelles relative to the focal plane, thereby providing an additional dimension of 3D morphological perception. Furthermore, the study highlights the potential for correlative imaging between iISM and fluorescence ISM. While the fluorescence channel provides molecular specificity for actin, the iISM channel leverages its superior sensitivity to reveal a panorama of filopodia and surrounding label-free structures that were previously invisible. This complementarity offers a novel observation window for dissecting complex intracellular interactions under near-physiological conditions.

As the first experimental validation of interferometric ISM, this work not only confirms the feasibility of integrating coherent scattering signals into the ISM architecture but also establishes a new paradigm for label-free super-resolution imaging. Diverging from single-modality approaches, the strategy of combining label-free interference with fluorescence detection enables the acquisition of multi-dimensional, complementary information. Such high-quality correlative data provides a robust foundation for intelligent image analysis. Looking forward, the integration of iISM with smart algorithms, such as event-triggered sensing, is expected to enable long-term, multi-dimensional dynamic analysis of live cells under conditions of extremely low phototoxicity.

## References

[1] Laissue, P. P. et al. Assessing phototoxicity in live fluorescence imaging. *Nat. Methods* **14**, 657–661 (2017). 28661494 10.1038/nmeth.4344

[2] Küppers, M. & Moerner, W. E. Interferometric image scanning microscopy for label-free imaging at 120 nm lateral resolution inside live cells. *Light Sci. Appl.* **15**, 129 (2026). 41748565 10.1038/s41377-026-02210-y PMC12946308

[3] Sheppard, C. J. R., Mehta, S. B. & Heintzmann, R. Superresolution by image scanning microscopy using pixel reassignment. *Opt. Lett.* **38**, 2889–2892 (2013). 23903171 10.1364/OL.38.002889

[4] Müller, C. B. & Enderlein, J. Image scanning microscopy. *Phys. Rev. Lett.* **104**, 198101 (2010). 20867000 10.1103/PhysRevLett.104.198101

[5] Roth, S. et al. Optical photon reassignment microscopy (OPRA). *Opt. Nanosc.* **2**, 5 (2013).

[6] De Luca, G. M. R. et al. Re-scan confocal microscopy: scanning twice for better resolution. *Biomed. Opt. Express* **4**, 2644–2656 (2013). 24298422 10.1364/BOE.4.002644 PMC3829557

[7] York, A. G. et al. Instant super-resolution imaging in live cells and embryos via analog image processing. *Nat. Methods* **10**, 1122–1126 (2013). 24097271 10.1038/nmeth.2687 PMC3898876

[8] Azuma, T. & Kei, T. Super-resolution spinning-disk confocal microscopy using optical photon reassignment. *Opt. Express* **23**, 15003–15011 (2015). 26072856 10.1364/OE.23.015003

[9] York, A. G. et al. Resolution doubling in live, multicellular organisms via multifocal structured illumination microscopy. *Nat. Methods* **9**, 749–754 (2012). 22581372 10.1038/nmeth.2025 PMC3462167

[10] Schulz, O. et al. Resolution doubling in fluorescence microscopy with confocal spinning-disk image scanning microscopy. *Proc. Natl. Acad. Sci. USA* **110**, 21000–21005 (2013). 24324140 10.1073/pnas.1315858110 PMC3876259

[11] Castello, M. et al. A robust and versatile platform for image scanning microscopy enabling super-resolution FLIM. *Nat. Methods* **16**, 175–178 (2019). 30643212 10.1038/s41592-018-0291-9

[12] Liang, Q. X. et al. High-fidelity tissue super-resolution imaging achieved with confocal2 spinning-disk image scanning microscopy. *Light Sci. Appl.* **14**, 260 (2025). 40754531 10.1038/s41377-025-01930-x PMC12319088

[13] Ren, W. et al. Expanding super-resolution imaging versatility in organisms with multi-confocal image scanning microscopy. *Natl. Sci. Rev.* **11**, nwae303 (2024). 40040644 10.1093/nsr/nwae303 PMC11879394

[14] Liao, J. H. et al. Deep-MSIM: fast image reconstruction with deep learning in multifocal structured illumination microscopy. *Adv. Sci.* **10**, 2300947 (2023). 10.1002/advs.202300947 PMC10520669 37424045

[15] Ingaramo, M. et al. Richardson–Lucy deconvolution as a general tool for combining images with complementary strengths. *ChemPhysChem* **15**, 794–800 (2014). 24436314 10.1002/cphc.201300831 PMC3986040

